# Image based magnetic field background correction for aortic and pulmonary artery flow measurement using phase contrast

**DOI:** 10.1186/1532-429X-14-S1-T12

**Published:** 2012-02-01

**Authors:** Joshua Yang Cheng, Yi Wang, William Schapiro, Nathaniel Reichek, Jie J Cao

**Affiliations:** 1 Research, St. Francis Hospital the Heart Center, Greenvale, NY, USA; 2 Bio-Medical Engineering, SUNY Stony Brook University, Stony Brook, NY, USA

## Background

Static magnetic field induced phase offset is common in phase contrast imaging and can cause significant errors in flow assessment. We sought to systematically assess phase shift in phantom experiments and validated methods of phase offset correction in human evaluation.

## Methods

A large gel phantom was used to assess phase offset in through-plane phase contrast images in x (left to right), y (anterior to posterior) and z directions at the isocenter and in positions ±40 mm and ±80 mm away from the isocenter. Repeated measures were averaged and fit to linear regressions. Phase correction was validated in human study for the assessment of aorta and pulmonary artery (PA) flow which was followed immediately by a phantom scan using the same settings. The aorta and PA flow corrected by the regression calculated background phase was compared to that corrected by the measured background phase in the phantom study.

## Results

The gel phantom experiments showed a linear phase descent in z direction through the isocenter with an average slope of -0.019±0.001 cm/s/mm (R2=0.998), demonstrating a velocity descent per millimeter away from the isocenter. The change in z direction was consistent in anterior, posterior, left and right sides of the phantom. There was also a linear phase descent (slope 0.009±0.0005 cm/s/mm, R2 =0.999) in the y direction but phase shift was limited in the x direction (slope 0.0005±0.0008 cm/s/mm, R2 =0.241). In human studies (N=30), average background phase velocity measured by phantom was 0.85± 0.56 cm/s in aortic position and 1.0±0.74 cm/s in PA, resulting in 8.9% and 7.5% flow errors in aorta and PA, respectively. The calculated aortic background phase velocity using linear regression was 0.81±0.47 cm/s which was not significantly different from phantom measured phase (p=NS). Calculated phase in the aortic position was applied to correct background phase in both aorta and PA flow given their proximity. Compared to phantom corrected measurements, the flow corrected using calculated phase yielded a very small residual error, mean 0.9±4.0 ml/beat in aorta and 0.9±5.1 ml/beat in PA.

## Conclusions

Phantom experiments demonstrated linear phase variations in the y and z directions but to a less extent in the x direction. Findings in human studies indicate that the linear phase shift is a significant source of phase error and can be removed using linear regression calculated phase from the image itself. Image based background phase correction is a promising alternative to phantom calibration in clinical practice.

## Funding

None.

